# Unsupervised Learning for Depth, Ego-Motion, and Optical Flow Estimation Using Coupled Consistency Conditions

**DOI:** 10.3390/s19112459

**Published:** 2019-05-29

**Authors:** Ji-Hun Mun, Moongu Jeon, Byung-Geun Lee

**Affiliations:** School of Electrical Engineering and Computer Science, Gwangju Institute of Science and Technology, Gwangju 61005, Korea; jhm@gist.ac.kr (J.-H.M.); mgjeon@gist.ac.kr (M.J.)

**Keywords:** unsupervised learning, depth estimation, camera ego-motion, optical flow, coupled consistency conditions

## Abstract

Herein, we propose an unsupervised learning architecture under coupled consistency conditions to estimate the depth, ego-motion, and optical flow. Previously invented learning techniques in computer vision adopted a large amount of the ground truth dataset for network training. A ground truth dataset, including depth and optical flow collected from the real world, requires tremendous effort in pre-processing due to the exposure to noise artifacts. In this paper, we propose a framework that trains networks while using a different type of data with combined losses that are derived from a coupled consistency structure. The core concept is composed of two parts. First, we compare the optical flows, which are estimated from both the depth plus ego-motion and flow estimation network. Subsequently, to prevent the effects of the artifacts of the occluded regions in the estimated optical flow, we compute flow local consistency along the forward–backward directions. Second, synthesis consistency enables the exploration of the geometric correlation between the spatial and temporal domains in a stereo video. We perform extensive experiments on the depth, ego-motion, and optical flow estimation on the Karlsruhe Institute of Technology and Toyota Technological Institute (KITTI) dataset. We verify that the flow local consistency loss improves the optical flow accuracy in terms of the occluded regions. Furthermore, we also show that the view-synthesis-based photometric loss enhances the depth and ego-motion accuracy via scene projection. The experimental results exhibit the competitive performance of the estimated depth and the optical flow; moreover, the induced ego-motion is comparable to that obtained from other unsupervised methods.

## 1. Introduction

Estimating accurate scene depth, ego-motion, and optical flow is a challenging issue in autonomous driving and robotics. Moreover, these properties are important in computer vision. Some components have extensive industrial applications, such as intelligence robotics [1] and simultaneous localization and mapping (SLAM) [2]. Typically, human eyes can easily detect an ego-motion and the direction of a scene in a short timescale. However, the creation of a model for real-world scene reconstruction encountered challenges of non-rigidity, occlusion, and light reflectance in past studies. Reconstructing a relevant model, despite these obstacles, depends on visual experiences, such as motion and the shapes of some specific objects. Particularly, we require a precisely predicted depth, because it provides crucial information to computer vision applications, such as driving assistance, object tracking, and three-dimensional (3D) reconstruction. Photometric-based depth estimation methods are mainly divided into two parts: stereo scenes and monocular scenes. Previous works replicated the binocular human vision system [3] using the support provided by a GPU for real-time processing to accurately estimate a depth in a short time from these source scenes [4]. However, they still suffered from a low depth quality with respect to the image scale divergence, occlusion area, and inconsistent luminance conditions.

Predicting a structure or motion from sequential scenes is a well-organized solution, and it is occasionally applied to unordered scenes. The traditionally used structure from motion (SfM) method [5] rapidly constructs a scene structure while using ego-motion. This technique is extremely sensitive, matches the camera position with the color scene, and deals with homogeneous regions. Recently, among the learning approaches, we have accessed numerous works on visual odometry techniques [6,7]. The part of the ground truth dataset that us used to train network structures was employed to perform odometry by these previous methods. Typically, extracting the feature points, matching them throughout the scenes, and then geometrically verifying the correctness of the matched feature points is the technique used to initiate a used process [8]. In a matching-points-based camera pose, reconstruction procedure and bundle adjustment [9] techniques are adopted to optimize the visual reconstruction. However, these approaches are vulnerable to geometrical restrictions, such as occlusions, complex textures, and homogeneous regions when extracting the feature points.

### 1.1. Supervised Learning

For several years, learning approaches have been widely used for feature matching and camera pose estimation. Camera ego-motion prediction can also be dealt using a supervised approach that is similar to that for the above-mentioned pixel-level depth prediction problem. The authors in [6] attempted to learn an informative visual feature from the input scenes using a convolutional neural network (CNN) to precisely extract the feature points. In addition, they were keen to learn the features during the ego-motion prediction and those that could predict a precise camera motion matrix from different viewpoint scenes. Estimating a camera pose and relocalization are directly dealt by learning tasks by considering the camera geometry [10]. Some authors [11] proposed a recursive CNN model targeting sequential scenes to enhance the accuracy of the feature extraction performance from a single scene. The pre-trained CNN achieved significant improvement than other feature extraction methods, such as handcrafted feature extraction [12]. The concept of probability, which is known as a conditional random field (CRF), can be applied to significantly improve motion estimation [13].

CNN architecture was adopted in [14] to estimate depth from a monocular scene. In addition, to estimate a depth from a calibrated stereo scene, [7] proposed a multi-scale network structure and scale invariant loss function for the network training. A similar work [15] estimated two-dimensional (2D) stereo target scenes from three-dimensional (3D) stereoscopic scenes. However, using a deep network structure causes a bottleneck and gradient vanishing problem. A residual-network-based fully convolutional architecture was proposed to model the relationship between a monocular scene and the depth map to tackle these limitations in a conventional CNN architecture [16]. Moreover, a deep feature extraction for depth estimation that optimized the cost volumes based on the stereo cost aggregation method was developed, because conventional stereo matching algorithms adopt the concept of cost volumes [17]. They regularized the matching cost volume to determine the depth from outdoor driving scenes. However, the aforementioned supervised methods for estimating depth, ego-motion, and optical flow required a precisely adjusted dataset. In addition, the network showed a different efficiency, depending on the type of the training dataset. Hence, these fully supervised approaches are vulnerable to the bias of the training dataset and they are easy to block in trivial solutions. Moreover, collecting numerous ground truth datasets from the real world requires significant human effort and time. The obtained dataset is still prone to natural noise and camera distortion although research on obtaining a precise dataset is conducted. A light detection and ranging (LIDAR) sensor sparsely records the distance from the sensor to an object; therefore, it is difficult to match its image to a colored image that is captured by a normal 2D camera. The structured light sensor suffers from the noise issues that are caused by light reflectance and dark region. Recent works have focused on an unsupervised approach, which could visualize a feature organization and be applied to extended vision tasks, such as depth estimation and scene reconstruction, to decrease some of the difficulty in obtaining a ground truth dataset.

### 1.2. Unsupevised Learning

In a recent work, researchers developed a learning framework with an unsupervised scheme. For some time, the camera geometric problem was considered to be unsolvable using a learning technique. However, its solution could be a neural network when utilizing an unsupervised learning technique. Various unsupervised approaches for the understanding of 3D works have been studied. The scene reconstruction concept was extended to model the loss function via the spatial smoothness in sequential scenes [18]. Similar to the depth estimation approach, photometric discrepancy was also considered in ego-motion prediction from a monocular scene [19]. The depth and camera ego-motion were induced throughout an unsupervised learning approach while using a monocular video, as exhibited in [20]. This work was very similar to our method, except for the fact that it obtained the depth and ego-motion from a monocular video scene. Other methods have attempted to build an efficient loss function by considering both the left and right consistency in a monocular depth estimation task [21]. These strategies considered whole pixels as equivalent 2D geometric properties, but this assumption was vulnerable to the homogeneous and occluded regions during network training. A binocular stereo scene with an unsupervised method [14] can train a network by minimizing the photometric discrepancy between the synthesized scene and the source scene to accurately predict the depth. In [22], the estimation of the camera pose and depth was conducted from monocular video sequences while using a Kalman filter. A Kalman filter can be included in the learning framework to improve the smoothness of the estimated camera ego-motion. In addition, the effort can be directed to avoiding fault aliasing problems by using a multi-stream CNN architecture.

### 1.3. The Contribution of This Work

**Figure 1:**
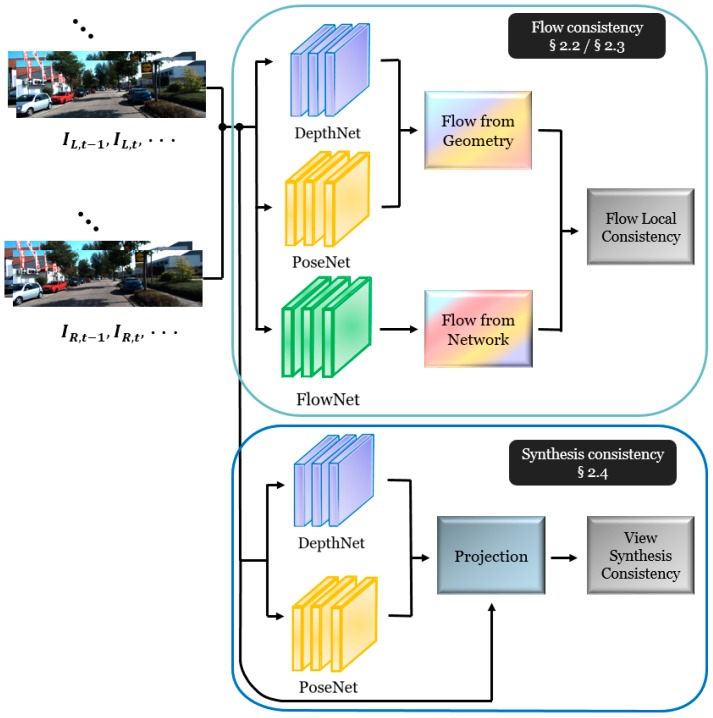
Flowchart of the proposed depth, ego-motion, and flow estimation.

Recent research topics on learning techniques for determining a 3D characteristic inference from linear 2D scenes motivate our work. Particularly, we propose an unsupervised-learning-based end-to-end training architecture to predict the depth, camera ego-motion, and optical flow from stereo sequences, as illustrated in Figure 1. The depth, ego-motion, and optical flow are simultaneously generated through the DepthNet, PoseNet, and FlowNet, respectively. Moreover, we describe the coupled consistency conditions by defining the following two consistency blocks, to enhance the accuracy of the estimated depth, ego-motion and optical flow values: flow consistency (Section 2.2) and synthesis consistency (Section 2.4) with a temporal variation and spatial with temporal variation in sequential scenes, respectively.

In the flow consistency block, we estimate a depth map from monocular sequential scenes and induce an ego-motion matrix with variation in the temporal domain. We simultaneously predict the optical flow from the depth plus camera ego-motion and FlowNet. We assume that the predicted optical flow values from the two different approaches have to correspond to each other at the pixel level. We project a target pixel to a source viewpoint scene to compute the photometric inconsistency between them to quantify the similarity. In addition, we propose a flow local consistency (Section 2.3) measurement method that penalizes the occluded regions while exploring a geometrical- and learning-based optical flow along the forward–backward direction to deal with the occlusion problem that is caused by the projection process. Even though the depth map and ego-motion are precisely estimated by the above-mentioned unsupervised approach, the projected scene from a target to a source is not exactly matched. In addition, it also includes the occlusion and discontinuity regions near the edge of the objects, owing to scene projection. We design our framework to train itself while computing the photometric dissimilarities between the spatially and temporally variant stereo videos in the synthesis consistency block to minimize these types of errors. Finally, we perform extensive experiments for a comprehensive evaluation of our proposed network architecture on the KITTI dataset [23]. Our coupled unsupervised approach provides a competitive result versus conventionally invented methods and it yields plausible results when compared to those that were obtained by a supervised approach.

The remainder of this paper is organized, as follows: A detailed explanation of the proposed method is provided in Section 2. In Section 3, we discuss the implementation details and experimental results. Finally, Section 4 presents the conclusion.

## 2. Method

This section explains our proposed framework of joint learning in sequential stereo scenes for depth, camera ego-motion, and optical flow prediction. Our method includes two main contributions: (1) a flow consistency block that measures the differences in the estimated flows that are obtained from the depth plus ego-motion and flow networks and (2) a synthesis consistency block that computes the photometric discrepancy in a synthesized view along the spatial and temporal domains.

### 2.1. Overview of Method

The key concept of our framework is to intensify the depth plus ego-motion and flow prediction network using different types of data comparison. The proposed framework trains the network while using sequential stereo scenes to estimate the depth, optical flow, and ego-motion without a paired dataset. We train each network while sharing the parameters under coupled consistency conditions. The flow consistency block generates a flow using depth plus ego-motion and a standalone flow estimation network, called FlowNet. The generated optical flow from the depth plus ego-motion is compared with the network-based predicted optical flow to penalize the photometrically dissimilar regions. Moreover, the flow local consistency measurement is newly proposed to exclude the occlusion effects along the forward–backward direction.

We measure the photometric consistency by projecting a target scene to a source viewpoint scene in the synthesis consistency block with the same depth and motion estimation network that was applied in the flow consistency framework. Using sequential stereo scenes facilitates comparison tasks in the spatial and temporal domains. Similar to the flow consistency framework, the photometric discrepancy was also measured along the forward–backward direction to avoid training inaccuracy in rigid regions, such as occlusion and discontinuous regions.

### 2.2. Flow Consistency with Depth and Ego-Motion

We build a flow consistency block that estimates a depth from a monocular video. A recently developed depth estimation method, while using a monocular scene [24], adopted a neural network on the supervision concept. However, the networks have to be trained with some part of the ground truth dataset, owing to the characteristics of supervised learning technique. However, our framework is working with an unsupervised scheme without any preliminary knowledge.

**Figure 2:**
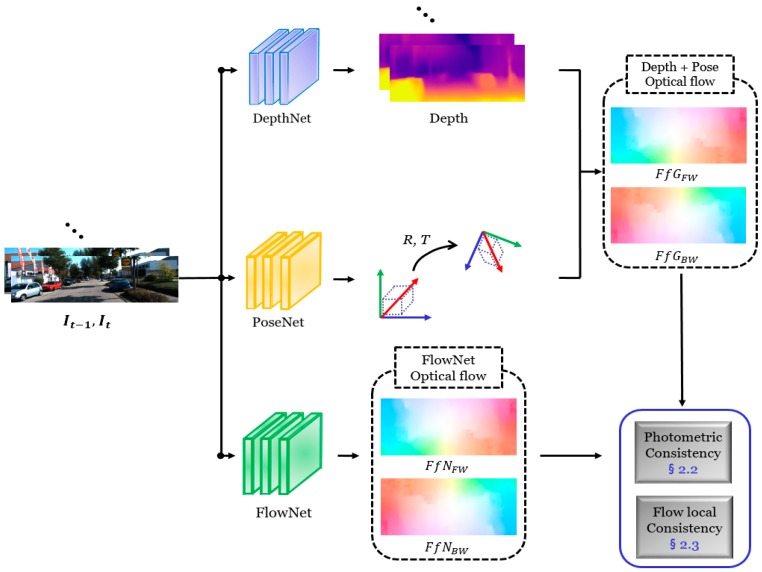
Flow prediction framework. The proposed architecture consists of two main parts: geometrical flow and network flow for the cross-check of the flow.

The objective of the flow consistency block is precisely estimating a depth and camera ego-motion while training a network using a monocular video, as illustrated in Figure 2. The network for estimating a geometrical camera ego-motion is differentiable, such that it is possible to perform gradient propagation for training CNN. In addition, we apply a generative adversarial network (GAN) [25] to produce a depth map from the monocular scene. We share the network weight values while training these consistency blocks, because the same depth estimation network is used in the flow consistency and synthesis consistency (Section 2.4) structure.

We train our depth prediction network that is based on GAN with cycle loss, as proposed in [26]. The problem of finding a global optimal solution from GAN can be replaced with training the network well. While training a network, we have to generate data $p_{g}$ and depth data $I_{D}$ having the same distribution. Based on this concept, we can define the training loss function for a depth estimation as (1). In this equation, $I_{C}$ and $I_{D}$ denote the color and depth domain distribution, respectively, and $I_{c}$ and $I_{d}$ denote the color and depth domain sample, respectively. Last, G and D represent the generator and discriminator that are used in each different color and depth domain.

(1) $$\underset{G}{\min}\;\underset{D}{\max}\;L_{I_{C}\to I_{D}}\left(G,D\right)=E_{I_{d}\sim I_{D}}\left[logD_{I_{D}}\left(I_{d}\right)\right]+E_{I_{c}\sim I_{C}}\left[log(1-D_{I_{D}}\left(G_{I_{C}\to I_{D}}(I_{c}\right)\right]$$

To determine a global optimum solution of (1), we assume the generator G is fixed with the optimal discriminator D, which can be represented as $D_{G}^{\prime }=I_{D}/(I_{D}+p_{g})$, according to that defined in [25]. Additionally, we consider that the distributions of the generator and color domain are the same ($p_{g}=I_{D}$). First, we solve (1) with respect to $\underset{D}{\max}$, so that it can be summarized as (2).

(2) $$\underset{G}{\min}L_{I_{C}\to I_{D}}\left(G,D\right)=E_{I_{d}\sim I_{D}}\left[log{D_{G}}^{\prime }\left(I_{d}\right)\right]+E_{I_{d}\sim p_{g}}\left[\log(1-{D_{G}}^{\prime }\left(I_{d}\right)\right]$$

In addition, $D_{G}^{\prime }$ has a value of 1/2, because we assume $p_{g}=I_{D}$. Furthermore, based on the Kullback–Leibler (KL) divergence and Jensen–Shannon divergence (JSD), (2) can be summed up as (3).

(3) $$\begin{array}{cc} \underset{G}{\min}L_{I_{C}\to I_{D}}\left(G,D\right) & =-\log\left(4\right)+\mathrm{KL}\left(I_{D}\|\frac{I_{D}+p_{g}}{2}\right)+\mathrm{KL}\left(p_{g}\|\frac{I_{D}+p_{g}}{2}\right)\\ & =-\log\left(4\right)+2\cdot \mathrm{JSD}(I_{D}\|p_{g}) \end{array}$$

Particularly, the JSD is 0 when the distributions $p_{g}$ and $I_{D}$ are the same. According to this characteristic, we notice that when the color domain and generator have the same distribution $p_{g}=I_{D}$, Equation (1) will reach the global optimum. Thus, the generator, G, should attempt to create maximally similar data with the depth domain data while training the adversarial network via the training dataset.

However, a conventional issue in GAN is the paradox between the min–max game and objective of the generator causing the problem of mode collapse owing to the adversarial objective fail to yield the correct solution under an isolated condition. We adopted a cycle consistency [26] that reached the global optimal solution under strictly limited conditions with (4) instead of using the original adversarial network standalone. The cycle consistency consisted of two generators in the color and depth domains, such as $G_{CD}\;:\;I_{C}\to I_{D}$ and $G_{DC}\;:\;I_{D}\to I_{C}$, and they work as a bijection mapping function. The translation of the cycle is directed to recover the translated data returning to the original domain. For example, data $I_{c}$ in the color domain C, can be recovered by the cycle condition, i.e., $I_{c}\to G_{CD}\left(I_{c}\right)\to G_{DC}\left(G_{CD}\left(I_{c}\right)\right)$. Similar to this scheme, depth data $I_{d}$ in the depth domain D, also adopts the cycle consistency, $I_{d}\to G_{DC}\left(I_{d}\right)\to G_{CD}\left(G_{DC}\left(I_{d}\right)\right)$. Due to the cycle consistencies that are used in our architecture attempting to minimize the pixel level discrepancies between the color and depth domains, they are efficient when we estimate a precise depth from a real world dataset composed of unpaired color and depth images.

(4) $$L_{Gycle}\left(G_{CD},G_{DC}\right)=E_{I_{c}\sim I_{C}}\|G_{DC}\left(G_{CD}\left(I_{c}\right)\right)-I_{c}{\|}_{1}+E_{I_{d}\sim I_{D}}\|G_{CD}\left(G_{DC}\left(I_{d}\right)\right)-I_{d}{\|}_{1}$$

By combining the GAN loss (1) and cycle consistency (4), we can define our objective loss function for DepthNet as (5). Our DepthNet estimates the accurate depth map without the biased problem in the real-world training dataset because the cycle consistency assists in avoiding GAN to experience the mode collapse problem.

(5) $$L_{CGAN}=L_{I_{C}\to I_{D}}\left(G,D\right)+L_{I_{D}\to I_{C}}\left(G,D\right)+L_{Gycle}\left(G_{CD},G_{DC}\right)$$

To project a pixel in consecutive video frames $I_{t-1}$ and $I_{t}$, we define the pixel level depth $D_{t}$, and the corresponding 4 × 4 camera rotation and transformation matrix as $T_{t-1\to t}$, which is an element of *SE*(3) (i.e., the special Euclidean group representing 3D rotations and translations). The 3D vectors in the transformation matrix, where u∈SO(3) indicates the rotation and translation vectors, are defined as $v\in {\mathbb{R}}^{3}$. We exploit the estimated depth and ego-motion for pixel projection to determine the corresponding coordinate between the source and target scenes. The projected pixel coordinate on source scene $p_{t}$ we will be determined by using the camera intrinsic parameter, K, and previously estimated depth and ego-motion with the target pixel coordinate, $p_{t-1}$. Subsequently, we can define the projected source viewpoint coordinate, $p_{t}$, as (6) based on [20].

(6) $$p_{t}=KT_{t-1\to t}D_{t}\left(p_{t-1}\right)K^{-1}p_{t-1}$$

Note that we did not perfectly match the projected pixel, $p_{t}$, with a coordinate of the source scene, owing to the inaccurate depth and ego-motion. Thus, we applied the differentiable bilinear interpolation technique [27]. This method linearly interpolates four neighboring pixels that are located near the inaccurately projected pixel coordinate. Next, we considered the photometric consistency loss by computing the pixel differences between the projected target scene and the source scene in entire 2D space, as defined in (7).

(7) $$L_{PF}=\underset{s}{\sum }\underset{t}{\sum }\left|I_{s}\left(p_{t}\right)-{\hat{I}}_{s}\left(p_{t}\right)\right|$$

where s and t are the source and target scene in the video data, respectively, and ${\hat{I}}_{s}$ represents the generated source scene by projecting the target domain pixel values.

However, the linear projecting operation on the source generates the domain scene, which is still only valid in the non-occluded regions and is invalid in occluded regions. We analyzed image correlation using the L1-norm and SSIM [28] loss to alleviate such an inaccuracy issue in the projected domain scene. Note that researchers in [21] measured the similarity between the source, $I_{s}$, and projected source viewpoint scene, ${\hat{I}}_{t}$, using (8).

(8) $$L_{FO}=\frac{1}{N}\underset{t,\;s}{\sum }\alpha \frac{1-SSIM\left(I_{s},\;{\hat{I}}_{t}\right)\;}{2}+\left(1-\alpha \right)\|I_{s}-\;{\hat{I}}_{t}\|$$

where SSIM adopts a 3 × 3 window kernel instead of using the Gaussian kernel to avoid the Gaussian blurring artifact, α is set as 0.85, and N represents the total number of pixels. In addition, we proposed a depth smoothness loss, as given in (9), to facilitate a depth that can be locally smooth while preserving the object boundary region and filtering out the discontinuous artifacts.

(9) $$L_{DS}=\frac{1}{N}\underset{p_{t}}{\sum }\left[\left|\nabla_{x}D\left(p_{t}\right)\right|\cdot e^{-\left|\nabla_{x}I\left(p_{t}\right)\right|}+\left|\nabla_{y}D\left(p_{t}\right)\right|\cdot e^{-\left|\nabla_{y}I\left(p_{t}\right)\right|}\right]$$

where $\nabla_{x}$ and $\nabla_{y}$ are the gradient operators in x and y directions, respectively, |·| denotes the element-wise absolute value, and D represents the estimated depth from DepthNet. By applying the x and y directional gradients to the gradient of the estimated depth map as the weight factors, the erroneous depth values in the estimated depth map were smoothed out.

### 2.3. Flow Local Consistency

We exploited the estimated optical flows from depth plus ego-motion to supervise the consistency between the source and target scene via projection. The estimated optical flow was still vulnerable to the dynamic objects in the temporal domain, even though we applied the error alleviation method for the occlusion area, as in Equation (8). In addition, we assumed that the projected pixels from the target to the source scene had to correspond to each other. However, this assumption is not typically ensured, owing to the occluded regions.

**Figure 3:**
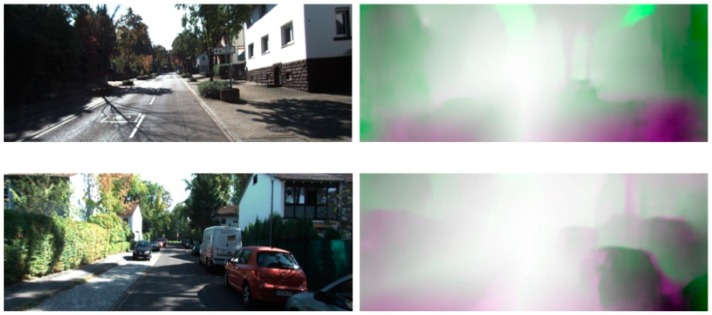
Flow local consistency check result. The **left** column scenes are the input color data, and the **right** column represents the local consistency results. The outside of each local consistency scene has more errors than the center of the result owing to scene variation.

We propose a flow local consistency (FLC) motivated by to address this problem [29]. This study defined a criterion that determined an invalid coordinate in terms of pixel differences. The forward and backward directional optical flows can be obtained from the depth plus ego-motion and FlowNet because we use monocular sequences. The FLC examines the similarity without modifying the structure of FlowNet. We measure the FLC by combining the bi-directionally estimated optical flow data, as defined in (10).

(10) $$L_{LC}=\underset{p}{\sum }\left|FfG_{FW}\left(p\right)-FfG_{BW}\left(p\right)\right|+\left|FfN_{FW}\left(p\right)-FfN_{BW}\left(p\right)\right|$$

where $FfG_{FW}$ and $FfG_{BW}$ represent the estimated flow from depth plus ego-motion along the forward and backward directions, respectively. Similarly, the flows from FlowNet are denoted as $FfG_{FW}$ and $FfN_{BW}$. Note that the pixel coordinate p, operates with all of the estimated flow information of the depth plus ego-motion FfG and FlowNet FfN. In Figure 3, we illustrate the investigated results of the FLC.

### 2.4. View Synthesis in Stereo Video

**Figure 4:**
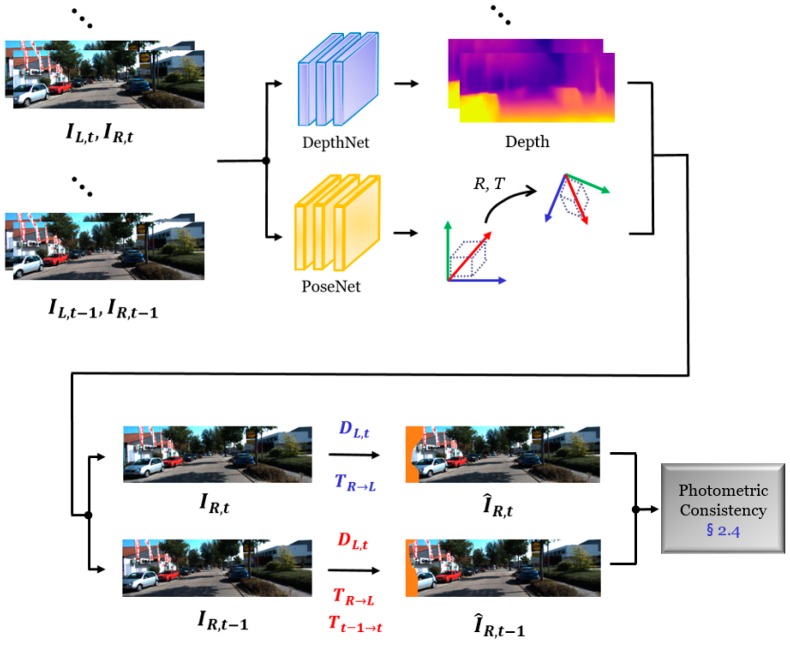
Detailed illustration of our proposed synthesis consistency block. Synthesized scenes from different target scenes are compared to the source scene to measure the photometric consistency in the spatial and temporal domains.

The training efficiency of our unsupervised network is highly related to the verification task of photometric consistency in sequential stereo scenes. As an extension of Section 2.2, we discuss how to enhance the performance of the depth and camera ego-motion estimation network by exploiting sequential stereo scenes. We used stereo sequences to compute the photometric discrepancies between the synthesized scene and source scene, as illustrated in Figure 4, contrary to the flow consistency block. We assumed that we knew the matrix between the source and target scene of a depth and camera ego-motion. The monocular-scene-based depth result had a problem in terms of the scale and rapid scene variation, even though we obtained the depth map via the depth estimation network.

Our framework used a stereo video for network training to overcome this problem. Instead of considering a scale ambiguity factor [19], we dealt with the issue using stereo consistency. Let a pair of stereo scenes in the temporal and spatial domains be $I_{L,\;t-1}$, $I_{R,\;t-1}$, and $I_{L,\;t}$, $I_{R,\;t}$. Among these scenes, we set the source and target scenes for view synthesis. From the predefined two target scenes, $I_{R,\;t-1}$ and $I_{R,\;t}$, we synthesized a source viewpoint scene by combining the estimated depth and the camera ego-motion. We adopted the same concept that was used in Equation (6) to synthesize the source scene from the target scenes. However, contrary to monocular scene projection, the stereo video had to use a different transformation matrix, owing to the variation in the geometrical characteristics in the temporal and spatial domains. Equations (11)–(13) exhibit different equations, depending on the domain of the target scenes. The source scene is synthesized using (11) when the source and target scenes are located in the same temporal domain but different spatial domains. If the source and target scenes do not exist in the same temporal and spatial domains, first, the target scene changes its spatial domain via (12). Subsequently, the translated target scene is converted to the source scene via (13).

(11) $$p_{R,\;t}=F\left(K,T_{R\to L},D_{L,t},\;p_{R,\;t}\right)$$

(12) $$p_{R,t-1}=F\left(K,\;T_{R\to L},\;D_{L,t-1},\;p_{R,t-1}\right)$$

(13) $$p_{L,t-1}=F\left(K,\;T_{t-1\to t},\;D_{L,t},\;p_{R,t-1}\right)$$

where F(·) represents the projecting function defined in (6) and $p_{R,\;t}$ and $p_{R,t-1}$ denote the pixel coordinates of the target scenes in different spatial and spatial–temporal domains, respectively. $T_{R\to L}$ and $T_{t-1\to t}$ are used to form the source scene from the target scenes, ${\hat{I}}_{R,t}$ and ${\hat{I}}_{R,t-1}$, respectively because our framework considers different viewpoint scenes for the photometric consistency measurement. The estimated translation matrix refers to the special Euclidian group and it is composed of a 4 × 4 matrix. This matrix is defined by six parameters, $T_{i\in \left\{x,\;y,\;z\right\}}$ and $r_{i\in \left\{x,\;y,\;z\right\}}$, where x, y, and z denote the three-dimensional coordinate values of the texture and depth scene. Finally, we set the loss function for synthesis consistency with photometric measurement, as Equation (14). ${\hat{I}}_{R,t}$ and ${\hat{I}}_{R,t-1}$ are the synthesized scenes that used the projected pixel coordinate from the target scenes with the spatial and temporal domains.

(14) $$L_{PV}=\underset{p}{\sum }\left(\left|I_{L,t}\left(p\right)-{\hat{I}}_{R,t}\left(p\right)\right|+\left|I_{L,t}\left(p\right)-{\hat{I}}_{R,t-1}\left(p\right)\right|\right)$$

The assumption of our synthesis consistency is that the scene projected in the source view point is Lambertian, such that the pixel intensity is constant, even though the spatial and temporal domains are variant. We consider that the synthesized scene as a key concept of depth and pose estimation using a CNN was already established in [20]. However, the authors had only synthesized the view in the temporal domain and not in the spatial domain. Note that the proposed synthesis consistency enforces DepthNet and PoseNet to exploit the relevant features. Therefore, we can mitigate the corrupted data propagated within the gradient operation in each network. In addition, the properties of the scene geometry enable the simultaneous concatenation of the depth and ego-motion by applying a different translation matrix ($T_{R\to L}$/$T_{t-1\to t}$) and depth map ($D_{L,t}$/$D_{L,t-1}$) for the spatial and temporal domain projections.

### 2.5. Loss Function for Training

We aimed to use the overall framework to build the unsupervised learning architecture for depth, ego-motion, and optical flow estimation under coupled consistency conditions while using flow consistency and view synthesis consistency. We have defined the parts of the training losses for the depth, ego-motion, and optical flow in Section 2.2, Section 2.3 and Section 2.4. We charged a penalty in terms of the dissimilarity in the photometric, occlusion, and flow local consistencies, because the estimated optical flow via the depth plus ego-motion network did not precisely correspond with the FlowNet results, particularly on the object boundary and occluded regions.

Furthermore, we set a specific loss function in the view synthesis scheme to enhance the robustness of the depth and ego-motion. With the spatial and temporal domain variations, we penalized the projection error that was caused by an inaccurate depth and ego-motion. Computing the photometric discrepancy between the synthesized and source scenes assisted our network to carefully predict the depth and ego-motion without the scale-variant, occlusion, and fast-moving object issues.

To summarize, our objective loss function in the proposed framework is defined, as follows:

(15) $$L=L_{FO}+L_{CGAN}+\lambda_{f}L_{PF}+L_{DS}+\lambda_{c}L_{LC}+\lambda_{v}L_{PV}$$

where $\lambda_{f}$, $\lambda_{c}$, and $\lambda_{v}$ represent the weight factors of the loss function in the photometric consistency of the flow, flow local consistency, and synthesis consistency, respectively. As the depth estimation network was trained in both the flow and synthesis consistency separately, we observe it in the entire training process.

## 3. Experimental Results

In this section, we present the verification of the efficiency of our proposed framework that was built using unsupervised learning under coupled constraint conditions. We evaluated our method on the KITTI dataset [23], which included prior works of depth, odometry, and optical flow. We performed an ablation analysis to certify the success of the proposed flow local consistency and spatial–temporal view synthesis under the constraint conditions in the network training, while changing the experimental conditions.

### 3.1. Dataset

We trained our framework with the KITTI dataset, which contained 61 raw video data with pre-processed stereo scenes. We divided the KITTI dataset in two parts to train our network in terms of depth and ego-motion.

We adopted the data splitting approach that was proposed by Eigen et al. [7] for a reasonable comparison of the conventional and proposed methods. Afterwards, we selected 33 stereo videos to train the network using 23,488 scenes. From the other 28 sequences, 697 scenes were used for training of the monocular depth estimation network.

However, for the odometry performance evaluation, we simulated the odometry data splitting method that was proposed in [20] while training the depth and motion network. The KITTI odometry data were composed of 11 sequences with the ground truth odometry data that were obtained via IMU/GPS sensors. We used the divided odometry sequences of 00–08 and 09–10 for the network training and evaluation, respectively. We fixed the length of the input sequence as 10 frames for the evaluation of our method. In addition, among the input stereo sequences of the divided odometry dataset, we set the target and source scenes as the sequential order of the input scene, such that the temporal relationship between the current and previous frames was well preserved.

### 3.2. Training Details

We implemented our framework using PyTorch [30] and performed all of the experiments using Intel i7-5960X. We trained the network on a single GTX 1080Ti GPU. We applied the Adam optimizer [31] with $\beta_{1}=0.9$ and $\beta_{2}=0.999$, learning rate 0.0001, and mini batch size 5 for the entire network training. While training the network, we used batch normalization [32] and ReLU activation [33] and followed the convolutional layers, except for the last one. The network-training task was converged within 150 epochs. We empirically found out the weights for loss functions $\lambda_{f}$, $\lambda_{c}$, and $\lambda_{v}$ set as 0.5, 0.3, and 0.2, respectively. This assisted in impacting the photometrical consistency rather than the other loss factors during the training process.

Our unsupervised coupled consistency architecture was composed of two core parts: flow consistency and view synthesis consistency. For DepthNet, which was modeled in the flow consistency block, we adopted the variant version of the convolutional residual network, ResNet50 [34], with cycling consistency [26]. We trained DepthNet using unpaired data to retain the property of an unsupervised scheme. We exploited DepthNet and PoseNet to deal with the consecutive stereo scenes in the spatial and temporal domains in the view synthesis consistency block. The output of PoseNet was six-degrees-of-freedom (6-DoF) vectors; in particular, the transform matrix had an element of SE3. We transformed the ego-motion vector to a concatenated form as a 4 × 4 matrix to evaluate the estimated ego-motion results.

### 3.3. Depth Estimation Results

**Figure 5:**
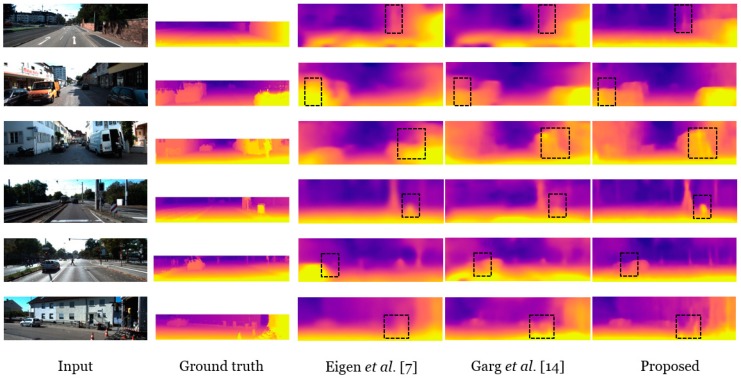
Comparison of the estimated monocular depth map results with the state-of-the-art supervised monocular depth estimation method results. Compared to Eigen et al. [5] and Garg et al. [8], our method shows more accurate depth results near the discontinuous region and object boundary.

We evaluated the performance of our depth estimation network with other state-of-the-art approaches while using the split KITTI dataset. As guided by [14], we set the range of the depth between 50 m and 80 m for the maximum threshold to evaluate the estimated depth errors. Figure 5 presents the estimated depth result samples.

As illustrated in the depth results, the proposed depth maps exhibit an improved result when compared to other methods [7,14] owing to GAN with cycling consistency [26] and the spatial–temporal correlations. Our method trained DepthNet using both monocular and stereo videos to satisfy the requirement of two different consistency conditions. In addition, in the view synthesis consistency, we could mitigate the occluded regions and discontinuity errors with the photometric loss of the ego-motion. Specifically, autonomous driving scenes cause occluded regions, owing to the difference between the frame rate and velocity of a vehicle. For instance, the KITTI dataset mainly has an occluded problem near the car, utility pole, and traffic sign. We indicate the improved depth accuracy of the occluded region using black dotted boxes in Figure 5.

In Table 1, we provide a quantitative comparison result of the depth maps that were obtained by our and conventional approaches. Moreover, the improved performance of our method is computed in terms of the average of all the error metrics and is then compared with conventional methods. The pioneer of learning-based depth estimation methods is Eigen et al. [7]. Through a stacked convolutional network for a coarse to fine depth generation method, they modeled multi-scale and scale-invariant loss functions. Even though [7] first introduced a neural network for depth estimation, they directly derived the depth value via a trained network on a supervised scheme without any insight of the 3D geometry properties. Therefore, our method shows a 24.42% improved performance than [7] with respect to scene geometry by newly proposing the FLC, which facilitates the exploration of the object boundary in forward–backward directions. Godard et al. [21] estimated depth from a monocular scene by exploiting the epipolar geometry constraints. However, their model estimated the pixel-level depth, from which harmful artifacts could be produced in the occluded regions owing to lack of the geometrical information. When compared to the result of [21], we achieve 5.74% enhanced depth accuracy because our method handles the occluded region in the optical flow and projecting operation. Garg et al. [14] measured the photometric error in a reconstructed source scene by an encoder–decoder structure, similarly to our approach. Although they dealt with the photometric consistency as a loss function, this was only in the spatial domain. However, our coupled consistency structure involves the photometric similarity factors in the spatial and temporal domains. Thus, we generate a 2.86% more accurate depth map than in [14]. Wang et al. applied the combined spatial loss functions (SSIM and L1-norm), being applied on the temporal domain to measure the photometric consistency [22]. They also removed unexpected pixels in the depth map using a left–right consistency mask. However, because our DepthNet is trained in the coupled consistency block with the depth smoothness loss, it shows a 7.12% improved depth map accuracy than [22]. Our proposed method outperforms the other depth estimation methods: [14] and [21], except for the Sq Rel and $\delta <1.25^{3}$ performance measurement metrics. Even though the conventional unsupervised methods of Zhou et al. [20] and Wang et al. [22] were trained on the same dataset as our architecture, our results exhibit a comparatively superior performance result. As the method presented in [20] mimicked a previously used architecture where learning the depth and ego-motion was connected, it did not consider the occluded region and motion of vigorous objects. By contrast, our method simultaneously considers these issues while also rigorously constraining the consistency condition in a coupled manner. Thus, our method can induce a 32.24% improved accurate depth map than [20].

In addition, we conduct ablation studies to prove the effectiveness of our joint consistency structure. When we use the trained network with the synthesis consistency structure, it shows an improved performance as compared to when we trained the network by only using the depth smoothness factor. When the depth smoothness term (9) is not included in the overall loss function, it shows a 10.53% and 6.59% worse result than conventional methods [14] and [21], respectively. Similar results are obtained when we only use the flow consistency (Section 2.2). However, when the synthesis consistency is involved in the loss function, it shows a comparable result to traditional methods. The view projecting operation, which changes the viewpoint from the target to the source scene, assists in identifying the relevant features in the spatial and temporal domains while estimating a depth map. Even though the synthesis consistency assists in improving the estimated depth accuracy, it still exhibits 9.82% worse performance than our coupled consistency condition. We note that the proposed coupled consistency architecture outperforms others in most of the quality measurement metrics. Particularly, the FLC reinforces the detection of the movement of dynamic objects while using bi-directionally generated optical flow data. In addition, the depth smooth term attempts to remove the error that occurs in the discontinuous regions.

### 3.4. Optical Flow Estimation Results

We compared our optical flow estimation accuracy with those of other conventional methods that adopted unsupervised and supervised schemes. We used the KITTI flow 2012 and 2015 datasets to verify the efficiency of the proposed method as compared to the other approaches.

**Figure 6:**
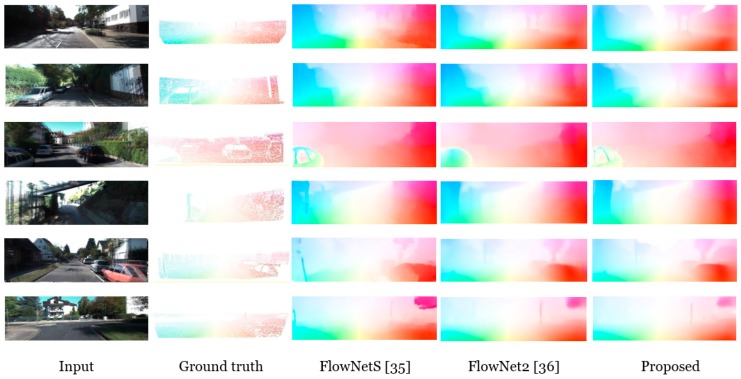
Visual optical flow evaluation results. Our networks are tested on KITTI flow 2012 and KITTI flow 2015. As shown in the flow estimation results, the proposed method clearly estimates the occluded and homogeneous flow values.

We computed the average end point error (EPE) with the ground truth optical flow that was provided by the KITTI dataset to evaluate our optical flow estimation result. In the case of the KITTI flow 2015 dataset, we additionally compute the average endpoint error (AEE) and Fl (training) score, which represent the ratio of the badly estimated value over three pixels and 5%, respectively, as compared to the ground truth in the estimated optical flow scenes. We compare the efficiency of the FLC method with the direct optical flow estimation method, which does not apply a consistency condition. As comparison methods of the optical flow experiments, we train the original FlowNet [35] and modified version FlowNet2 [36]. In addition, we test with a part of the network structure that was proposed by some researchers [35]: FlowNetS, which includes encoder–decoder architecture, and FlowNetC, which deals with correlated feature maps. They trained these networks using FlyingChairs [35] and FlyingThings [37]. The corresponding dataset consisted of rendered random scenes on a 3D model with variant motion and under lightning conditions. the proposed method achieves an improved optical flow accuracy near the object boundary when compared to the results of the other methods, as exhibited in Figure 6.

Table 2 lists the quantitative comparison results. Our flow estimation network can generate a precise optical flow, because our FLC attempts to exclude the occluded regions by penalizing the loss function throughout the forward–backward crosscheck. Therefore, our results outperform those of FlowNetS and FlowNetC, not only in terms of the Fl-score of 23.08% and 18.15%, respectively, but also in other error metrics in both the KITTI 2012 and KITTI2015 datasets. Similar to our approach, DSTFlow [38] is modeled under an unsupervised scheme. It computes the photometric similarity in each convolutional layer while training the network using an unpaired dataset. Particularly, it adopts the Ghabonnier [39] penalty composed of grey and gradient constancies. Even though it is devised using an unsupervised flow estimation method, it lacks 2D geometrical properties, such as occlusion and discontinuous regions, which causes the deterioration of the estimated flow quality. Note that, in our method, we deal with the geometrical limitations using the FLC loss function. Thus, the accuracy of our optical flow results with respect to the F1-score is 6.22% higher when compared to that of [38] method. Contrary to our method, the conventional method partially exploits the characteristics of the training data. Therefore, in KITTI 2012, FlowNet2 [36] shows a better performance than our method with respect to the EPE and non-occluded regions by 1.72% and 32.16%, respectively. It simultaneously deals with the warped data and small displacements in the scene. Thus, FlowNet2 marginally improves the accuracy of the optical flow as compared to our results. However, our results show enhanced performance in KITTI 2015 with a 0.59% Fl-score as well as remaining error metric. Even though our method is built in an unsupervised manner, it induces improved performance versus supervised-scheme-based methods, such as in [35,36].

Additionally, the running time of each algorithm was measured in milliseconds (ms). We excluded the training time and used the same number of test images to compare the runtime of each algorithm, as defined in Section 3.1. Even though our framework dealt with joint consistency conditions during the network training, it was more than twice faster than the recently invented flow estimation algorithm [36]. Owing to the jointly trained structure of our method with respect to the flow and synthesis consistency terms, its time duration was more than that of the conventional method [34]. However, our ablation experimental results showed that the proposed method has low computational complexity when compared to FlowNetC [35]. The running time of the flow estimation on a CPU [38] was not compared with our method.

### 3.5. Camera Ego-Motion Estimation Results

We evaluated the performance of the proposed ego-motion prediction network on the KITTI odometry dataset. We divided the 11 KITTI odometry datasets into two parts with the ground truth data. We used 09–10 to test the network, because our ego-motion network was trained using sequences 00–08. We compared the proposed method with a typically used SLAM algorithm, the ORB-SLAM [40] framework. The short version of ORB-SLAM contained five frames, and the long version of ORB-SLAM had all the frames. Five frames were used, for which the scaling factor was optimized with the ground truth data, in the evaluation of the trajectories of two versions. The loop-closure detection method was used for optimization. These two versions of ORB-SLAM adopted the bundle adjustment technique and fixed scale maps to track the trajectory. In addition to ORB-SLAM, we also compared supervised and trained state-of-the-art ego-motion estimation algorithms [20,41]. Particularly, Zhou et al. [20] estimated a camera pose by monocular learning with a frame-to-frame approach, and their trajectory was aligned with the ground truth data. The length of the frame for training was fixed as 5. Yin et al. [41] suggested an adaptive geometric consistency term to enhance the robustness on the outliers and the non-Labmertian area under an unsupervised scheme.

We evaluated the ego-motion accuracy by computing the absolute trajectory error (ATE). Or method shows a better performance than the conventionally used methods [20,40] by about 0.050 ± 0.126 and 0.0205 ± 0.016 on average, respectively, as listed in Table 3. We compute both the origin error and additional error range simultaneously in Seq. 09 and Seq. 10 to analyze the improved performance of ATE. As our pose estimation architecture is trained under both the flow and view synthesis consistency conditions, it achieves a better result even in the full version of ORB-SLAM. Our PoseNet continuously maintains the trajectory by adjusting the camera pose in the temporal domain. As indicated in the ablation study result, when we exploit the synthesis consistency for training the overall network, it shows improved results.

The recently invented unsupervised algorithm in [41] produced a precise trajectory result by dividing the rigid and non-rigid regions separately in the optical flow domain. In addition, they captured the high-level cues instead of the low-level feature matching points to enhance the detailed expression of active objects. This is the main reason why our approach exhibits a worse performance than the [41] method. Our method shows a degraded performance by 0.0015 ± 0.0015 with an averaged ATE in Seq. 09 and Seq. 10. We can modify our network to embrace the photometric and local consistency conditions in the spatial–temporal domain in the flow consistency block to improve the performance of our ego-motion network as compared to its present status.

## 4. Conclusions

We proposed an unsupervised learning approach while using coupled consistency conditions to estimate the depth, ego-motion, and optical flow from stereo sequences. Our core concepts were composed of two components, which trained the network by flow consistency and synthesis consistency. The flow consistency block attempted to minimize the discrepancy between the geometrically estimated optical flow and network-based optical flow. Moreover, the synthesis consistency block mainly inspected the photometric similarity of the source and synthesized scenes under a varying spatial–temporal domain. We verified the efficiency of our framework via extensive experiments on the KITTI raw, optical flow, and odometry dataset. Our depth estimation and optical flow architecture had improved results when compared to the conventional supervised approaches. In addition, the ego-motion accomplished promising achievements with the recently invented neural-network-based algorithms.

There are yet some remaining challenges that will have future solutions. Our method did not focus on the small motions in a video. Vehicle driving scenes, object tracking techniques, and sensing small motion information are other areas of research that are yet to be explored. We must design new models while dealing with the ego-motion information to detect a small motion in dynamic and rigid scenes under a learning approach. Furthermore, extensive research regarding the ego-motion estimation technique in an adaptively varying temporal domain is required.

## Figures and Tables

**Table 1:** Monocuar depth estimation result on KITTI dataset [23]. K denotes the KITTI dataset. (U) is unsupervised approach. (D) represents depth supervision, and (P) denotes pose supervision.

Method	Dataset	Error Metric				Accuracy Metric		
		Abs Rel	Sq Rel	RMSE	RMSE log	δ<1.25	$\delta <1.25^{2}$	$\delta <1.25^{3}$
Zhou et al. [20]	K (U)	0.208	1.768	6.856	0.283	0.678	0.885	0.957
Eigen et al. [7] Coarse	K (D)	0.214	1.605	6.563	0.292	0.673	0.884	0.957
Eigen et al. [7] Fine	K(D)	0.203	1.548	6.307	0.282	0.702	0.890	0.958
Godard et al. [21]	K (P)	0.148	1.344	5.972	0.247	0.803	0.922	0.964
Garg et al. [14]	K (P)	0.169	1.080	5.104	0.273	0.704	0.904	0.962
Wang et al. [22]	K (U)	0.154	1.333	5.996	0.251	0.782	0.916	0.963
Ours (w/o depth smooth)	K (U)	0.183	1.442	5.289	0.264	0.686	0.891	0.955
Ours (w/o synt. cons.)	K(U)	0.171	1.597	5.337	0.252	0.692	0.898	0.955
Ours (w/o flow cons.)	K (U)	0.158	1.514	5.293	0.271	0.694	0.888	0.951
Ours	K (U)	0.143	1.328	5.102	0.244	0.803	0.930	0.960

**Table 2:** Quantitative optical flow comparison result on the KITTI flow 2012 and KITTI flow 2015 datasets. The Flying Chairs dataset [35] is denoted as C, Flying Things dataset [37] as T, and KITTI raw dataset as K. The learning approach (G) is supervised by the ground truth, and (U) represents an unsupervised method. Non-Oc indicates a non-occluded region.

Method	Dataset	KITTI Flow 2012			KITTI Flow 2015				Runtime (ms)
		EPE (Train)	EPE (Test)	Non-Oc	EPE (Train)	AEE (Train)	Fl (Train)	Non-Oc	GPU
FlowNetC [35]	C (G)	9.35	-	7.23	12.52	-	47.93%	9.35	51.4
FlowNetS [35]	C (G)	8.26	-	6.85	15.44	-	52.86%	8.12	20.2
DSTFlow [38]	K (U)	10.43	12.4	-	16.79	14.61	36.00%	-	-
FlowNet2 [36]	C (G) + T (G)	4.09	-	3.42	10.06	9.17	30.37%	4.93	101.6
Ours (w/o flow cons.)	K (U)	5.33	4.72	6.21	11.28	10.11	35.42%	5.13	38.1
Ours (w/o synt cons.)	K (U)	5.02	4.55	6.69	10.74	9.58	34.17%	5.08	38.2
Ours (w/o FLC)	K (U)	4.41	4.38	5.45	10.33	9.37	31.64%	4.96	40.4
Ours	K (U)	4.16	4.07	4.52	10.05	9.12	29.78%	4.81	42.8

**Table 3:** Estimated ego-motion evaluation result. The performances are measured by computing the absolute trajectory error (ATE). The best performance is remarked in bold, and the second best is underlined.

Method	Seq. 09	Seq. 10
ORB-SLAM (full) [40]	0.014 ± 0.008	0.012 ± 0.011
ORB-SLAM (short) [40]	0.064 ± 0.141	0.064 ± 0.130
Zhou et al. [20]	0.021 ± 0.017	0.020 ± 0.015
Yin et al. [41]	0.012 ± 0.007	0.012 ± 0.009
Ours (w/o synt. cons.)	0.018 ± 0.026	0.017 ± 0.022
Ours	0.014 ± 0.009	0.013 ± 0.010
